# Estrogen independent gene expression defines clinically relevant subgroups of estrogen receptor positive breast cancer

**DOI:** 10.1186/1471-2407-14-871

**Published:** 2014-11-24

**Authors:** Robin M Hallett, John A Hassell

**Affiliations:** Department of Biochemistry and Biomedical Sciences, Centre for Functional Genomics, McMaster University, 1200 Main Street West, Hamilton, Ontario L8N 3Z5 Canada

**Keywords:** Breast cancer, Gene expression, Subtypes, Therapies, Estrogen

## Abstract

**Background:**

Human breast cancer represents a significantly heterogeneous disease. Global gene expression profiling measurements have been used to classify tumors into multiple molecular subtypes. The capacity to define subtypes of breast tumors provides a framework to enable improved understanding of the mechanisms of breast oncogenesis, as well as to provide opportunities for improved therapeutic intervention in patients.

**Methods:**

We used publicly available gene expression profiling data to identify ‘estrogen independent’ genes in estrogen receptor alpha (ER+) breast tumors, and subsequently identified 6 subgroups of ER + breast tumors.

**Results:**

Each of the 6 identified subgroups exhibited distinct clinical behaviors and biology. Patients whose tumors comprised subgroups 2,5&6 experienced excellent long-term survival, whereas those patients whose tumors belonged to subgroups 1&4 experienced much poorer survival. Breast tumor cell lines representative of the different subgroups responded to therapeutic compounds in accordance with their subgroup classification.

**Conclusions:**

These data support the existence of 6 distinct subgroups of ER + breast cancer and suggest that knowledge of the ER + subgroup status of patient samples have the potential to guide therapy choice.

**Electronic supplementary material:**

The online version of this article (doi:10.1186/1471-2407-14-871) contains supplementary material, which is available to authorized users.

## Background

There is significant molecular and cellular diversity among human breast tumors. Indeed, this heterogeneity is evident from histopatholologic features and differences in ER, progesterone receptor (PR) and ERBB2/HER2/NEU status as well as more recent molecular classification schemes based on the expression of large numbers of genes [1–3]. Importantly, these data indicate that breast cancer is an imprecise definition that embodies many molecularly distinct neoplastic disorders that share a common normal breast tissue origin.

The capacity to more accurately define breast cancers and identify tumor subgroups that represent more homogeneous disease entities, provides a framework to increase our understanding of these diseases and provides opportunities to focus treatment options for patients. To this end investigators have completed relatively large gene expression studies and identified patterns in gene expression that reproducibly stratify breast tumors into each of 5 molecular subtypes. These breast cancer subtypes named basal-like, ERBB2-positive, normal-like, luminal A and luminal B were originally described by Perou *et al*. [1]. The various molecular subtypes possess distinct clinical behaviors thus providing a basis for improved taxonomy for breast cancer. For example, basal-like tumors are highly aggressive, resistant to endocrine therapies but sensitive to conventional chemotherapy, whereas luminal A tumors are more indolent and responsive to endocrine therapies. Importantly, recent and more comprehensive molecular profiling of human breast tumors, including global gene expression, mutation, DNA copy number variation, and protein expression support the original finding that breast cancer falls into major molecular subtypes comprising subsets of genetic and epigenetic abnormalities [4]. Currently, the additional clinical value of molecular classification over traditional histopathological methods is unclear, as the molecular subtypes show high correspondence to the expression of ER, PR, and HER2, as well as to tumor grade [3].

It is possible that further refinement of the ‘intrinsic’ classification scheme of Perou *et al*., could identify other molecular classes of breast cancer, and provide additional clinical value beyond traditional techniques. For example, ER + tumors generally fall into the luminal A and B molecular subtypes, characterized by expression of the ER as well as cytokeratins typically expressed by luminal epithelial cells [1, 3]. However, more recent studies suggest that as many 12 molecular subgroups of ER + breast cancer exist, demonstrating that the luminal A and B stratification of ER + breast tumors does not fully capture the biological complexity of these tumors [5]. Indeed, further dissection of ER + breast tumors into additional relevant disease subgroups would likely provide further insight into the mechanisms that underlie these tumors, as well as prevent carefully planned studies from being confounded by the heterogeneity found among un-grouped or sub-optimally grouped populations of ER + breast tumors. Notably, the molecular subtypes of breast cancer show subtype specific response to standard chemotherapies as well as experimental compounds, highlighting the value of investigating specific disease subtypes [6]. Hence, the identification and characterization of additional subgroups of ER + breast tumors could focus treatment options for patients with ER + breast tumors, because therapy could be rationally applied based on specific molecular characteristics of the patient’s tumor.

We hypothesized that the biology of ER + tumors comprised both estrogen-dependent and -independent components, and furthermore, that investigation and characterization of the estrogen independent component might provide a means to stratify ER + tumors into different distinct disease subgroups. To this end we used publicly available data to identify ‘estrogen independent’ genes in ER + breast tumors and subsequently identified subgroups of ER + tumors based on molecular differences between tumors identified by these genes. Importantly, we reproducibly identified 6 subgroups of ER + breast tumors that exhibited distinct clinical behavior as well as biology. Moreover, we show that these subgroups have specific responses to therapeutic compounds *in vitro*. Taken together these data support the existence of 6 distinct subgroups of ER + breast cancer, and advance efforts to increase the precision of therapeutic intervention in human breast cancer patients.

## Methods

### Human breast tumor data sets

All tumor samples were downloaded from the gene expression omnibus (GEO, http://www.ncbi.nlm.nih.gov/geo/). The latter included Letrozole treated tumor samples (GSE5462) [7], the discovery cohort (GSE6532, 133A array samples, n = 327 [8], the validation cohort (GSE6532 133 Plus 2.0 array samples n = 87 [8], GSE9195 n = 77 [9], GSE17705 n = 298 [10], GSE2034 n = 209 [11], GSE7390 n = 134 [12], Original samples from GSE26971 (n = 136)). Cell line expression profiles were downloaded from ArrayExpress (E-TABM-157) [13]. Raw data files representing the tumor samples were normalised using RMA [14]. TCGA gene expression was obtained from the TCGA research network ( http://cancergenome.nih.gov/.), by downloading level 3 RNAseq data from the TCGA data portal (RSEM normalised) [15]. For GEO cohorts, ER + status was obtained from associated clinical files, which were generally based on histopathological assessment. ER + status for the TCGA cohort was determined using expression cut-offs (250 RSEM normalised transcript counts) for the ESR1 gene. ER + patients were selected from each dataset, and validation cohorts were combined after each probe set/gene was standardized and mean centered.

### Cell line drug sensitivity data

We obtained previously reported human breast tumor cell line sensitivity data from Heiser *et al*. [6].

### Definition of estrogen independent genes

We calculated within (w, treatment pairs) and between (b, independent primary tumor samples) variation for all tumors. In this fashion probe-sets with greater variation in expression between tumors than between treatment paired samples received high b/w scores, and vice versa.

### PAM 50 subtype assignment

Subtype membership was assignment was based on the nearest PAM50 centroid (Pearson correlation) [16].

### Class discovery

Non-negative matrix factorization was carried out as previously described [17]. Prediction analysis of microarrays (PAM) was carried out as described [18] to discover subgroup specific genes (discovery cohort) and to classify samples (validation cohort, cell lines).

### Cell growth assays

All cell lines were obtained from the ATCC and passaged minimally prior to completing these experiments. Cell lines were maintained as suggested by the ATTC. Cell lines were maintained in either RPMI or DMEM supplemented with 10% fetal bovine serum (all from Life Technologies). Cells were seeded at a density 50,000 cells/ml in the wells of a 6-well plate (Corning) in triplicate for each time point. At each time point cells were trypsinized and viable cells were counted with a hemocytometer using Trypan Blue exclusion as a marker of cell viability. Relative cell growth was calculated as a number of viable of cells relative to control at each time point.

### Statistical analysis

Survival analysis and Log-rank tests were used to evaluate survival differences between patient subgroups. We used 10 year distant metastasis free survival (DMFS) or disease free survival (DFS) as the clinical endpoint for these studies, and log-rank tests to detect differences in survival. T-test were used to compare means for 2-group comparisons, whereas ANOVA followed by Dunnett’s multiple comparison test was used to compare means for 3 or more groups. Tests were two-sided and a p-value of 0.05 or less was considered statistically significant.

## Results

### Identification of estrogen independent genes and distinct subgroups of ER + breast cancer

**Figure 1:**
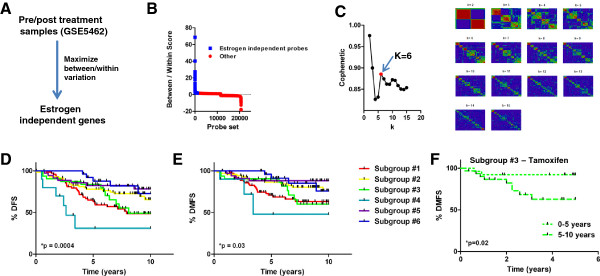
**Discovery of estrogen independent genes and subgroups. A)** Experimental strategy to identify estrogen independent genes. **B)** Between/within scores for all probe sets. **C)** NMF consensus analysis of discovery cohort identifies 6 subgroups of ER + breast tumors. **D)** Disease free survival analysis of the training cohort stratified into the 6 subgroups. **E)** Distant metastasis free survival analysis of the training cohort stratified into the 6 subgroups. **F)** Comparison of pre/post 5 year survival in tamoxifen treated subgroup #3 patients.

The goal of this study was to enable classification of ER + breast tumors on the basis of genes whose expression is related to the estrogen independent biology of ER + tumors. To this end, we took advantage of the gene expression profiles of 58 ER + breast tumors biopsied from post-menopausal women before and after treatment with Letrozole (n = 116, [7]). Because letrozole treatment induces estrogen deprivation in tumors of post-menopausal women, we hypothesized that genes whose expression showed minimal variation after letrozole treatment could be considered to be expressed independent of estrogen. To identify estrogen-independent genes that might be useful to identify subtypes of ER + breast tumors, we calculated between/within (b/w) scores for each probe set, which were measurements of probe set variation observed between different primary tumors relative to the variation observed within paired samples pre- and post-treatment. In this fashion, probe sets with high b/w scores showed greater variation between different primary tumors than between treatment paired tumor samples, whereas genes with low b/w scores showed greater variation within treatment paired tumors samples than between different tumors. Therefore, probe sets with high b/w scores were likely not influenced by estrogen deprivation and also showed variable expression among the different tumors prior to treatments, suggesting that they are related to differences in the estrogen independent biology of tumors (Figure 1A). We selected the top (highest b/w scores) 1,000 estrogen independent probe sets (893 genes) for further analysis (Figure 1B, Additional file 1: Table S1).

To investigate whether the expression of the estrogen independent probe sets could capture the phenotypic complexity of ER + breast tumors we completed unsupervised clustering using non-negative matrix factorization (NMF) [17]. NMF is an efficient method to identify molecular patterns that is readily applicable to gene expression data, and therefore can be used as a powerful means for class discovery. In short, NMF identifies metagenes, or distinct gene expression patterns, which are used to determine the optimal value for k, where k represents the number of sample subgroup clusters by calculating a cophenetic co-efficient for each value of k. In short, we applied NMF (for k = 2-10) to gene expression data representing 262 primary ER + breast tumors (GSE6532, 133A arrays, [19] filtered such that only the 1,000 estrogen independent probe sets were used for class identification. This data set optimally fell into 6 clusters, designated subgroups 1–6 (Figure 1C). Moreover, NMF on an additional independent data set of 298 ER + breast tumors (GSE17705, [10]) using the same 1,000 estrogen independent probe sets also suggested that these patients were also optimally stratified into 6 subgroups (Additional file 2: Figure S1). Hence, we concluded that on the basis of the expression of estrogen independent genes, ER + breast tumors can be categorized optimally into 1 of 6 ER independent subgroups.

To learn whether these groups might encompass disease with different clinical outcomes we compared DFS (Figure 1D, *p < 0.05, Log-rank test) and DMFS (Figure 1E, *p < 0.05, Log-rank test) among the various subgroups. Interestingly, some subgroups displayed excellent long term outcomes, whereas other groups did not. For example, 10 year DMFS in subgroup 5 patients was 88%, whereas in subgroup 4 patients it was 48%. All patients comprising the various subgroups were uniformly chemotherapy naïve, suggesting that these differences in survival are likely related to the natural progression of their disease, rather than influenced by response to chemotherapy. Interestingly, in tamoxifen treated subgroup #3 (n = 27) patients we observed that the majority of DMFS events occurred after 5 years (Figure 1F), the time at which most patients cease tamoxifen treatment, possibly suggesting that these patients would have benefited from tamoxifen treatment beyond 5 years. Unfortunately, this dataset only comprised 8 subgroup #3 patients who did not receive tamoxifen, making the complimentary analysis in tamoxifen naive patients impractical.

### Subgroups are independent of the molecular subtype of breast cancer

**Figure 2:**
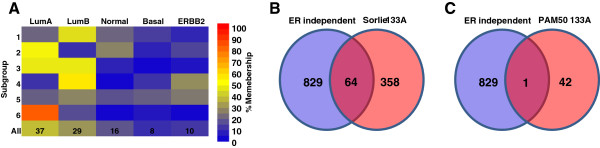
**6 subgroups classification is independent of tumor molecular subtype membership. A)** Subgroup/Subtype assignment of each tumor. **B)** Overlap between ER independent genes and intrinsic genes. **C)** Overlap between ER independent genes and PAM50 genes.

Significant data exists that breast tumors can be stratified into at least 5 molecular subtypes [1, 2, 16]. Accordingly, we examined whether there was an association between the 6 subgroups we identified and the 5 molecular subtypes of breast cancer. Classification of the 262 ER + tumors used for discovery using the PAM50 genes (43 genes present on 133A arrays), revealed that most of the tumors were classified into either the luminal A (37%) or luminal B (29%) molecular subtypes, whereas of the remainder 16% were normal, 8% were basal, and 10% were ERBB2 (Figure 2A, Additional file 2: Figure S2). Among the 6 ER independent subgroups, only subgroup #6 was strongly associated with any of the 5 molecular subtypes of cancer, and comprised ~82% Lum A, ~14% LumB, and ~4% Basal-like tumors (Figure 2A). Among the 893 ER + independent genes, only 64 overlapped with the Sorlie *et al*. intrinsic genes [2], and only 1 overlapped with the 43 PAM50 genes [16], present on the 133A array (Figure 2B and C). Hence, we concluded that the classification of ER + breast tumors into the 6 subgroups we identified was relatively independent of their membership in the 5 molecular subtypes of breast cancer.

### A framework for ER + breast tumor classification

**Figure 3:**
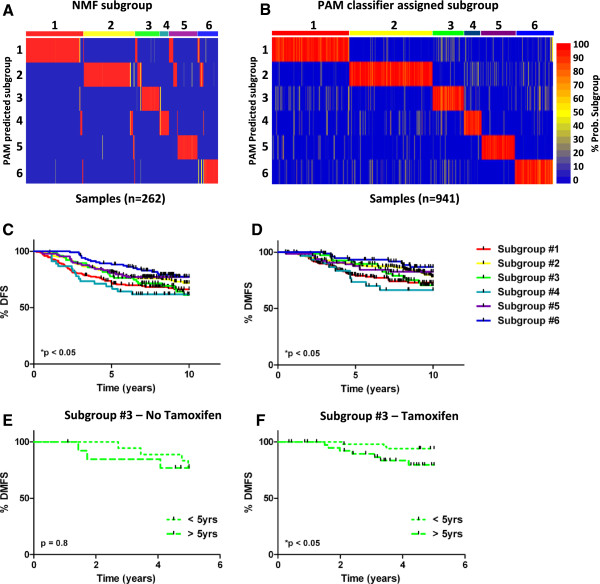
**The 6 subgroups are reproducibly identifiable. A)** Subgroup assignment for NMF or 300 probe set PAM classifier (83% concordance). **B)** PAM assignment of validation cohort tumors into the 6 subgroups. **C)** Disease free survival among the validation cohort patients stratified by subgroup. **D)** Distant metastasis free survival among the validation cohort patients stratified by subgroup. **E)** Comparison of pre/post 5 year survival in tamoxifen naive subgroup #3 validation cohort patients. **F)** Comparison of pre/post 5 year survival in tamoxifen treated subgroup #3 validation cohort patients.

To confirm that the 6 subgroups we identified were indeed generally representative of ER + breast tumors, we investigated their prevalence in an independent validation dataset comprising 941 ER + chemotherapy-naïve breast cancer patients. Briefly, we identified a 300 probe set classifier (using PAM, top 50 probe sets of each subgroup) to classify tumors into the 6 subgroups (Figure 2A, >80% concordance with NMF classification). Based on the expression of the 300 probe sets, we assigned each tumor comprising the validation data set in the 6 subgroups, using PAM (Figure 2B). Some 84% (n = 788) of the tumors in the validation set were assigned with a probability higher than 80% of belonging to one of the 6 subgroups, demonstrating that the classification framework is robust. Notably, the DFS and DMFS characteristics of patients comprising the various 6 groups were found to be highly coincident between the original (n = 262) and validation (n = 941) cohorts (Figure 3C and D, Additional file 2: Figure S3, Correlation: 0.89, *p < 0.05). For instance, 10 year DMFS was lowest in subgroup 4 for both the original and validation datasets. Similar to observations made in our training cohort, we observed that patients with subgroup #3 tumors experienced the majority of DMFS events after 5 years (Additional file 2: Figure S4). To learn whether this phenomenon was related to tamoxifen treatment, we subdivide subgroup #3 patients into tamoxifen treated (n = 94) and tamoxifen naive patients (n = 32) and compared pre/post 5 year DMFS survival in each group. Whereas there was no difference between pre/post 5 year DMFS in tamoxifen naive patients, there was a significant different pre/post 5 year DMFS in tamoxifen treated patients (Figure 3E and F, No tamoxifen, HR: 0.82, p = 0.8, Tamoxifen, HR: 0.26, *p < 0.05). These results might be interpreted to suggest that in subgroup #3 tumors early relapse is prevented by tamoxifen, albeit relapses resume after the completion of a patient’s tamoxifen regimen. Indeed, clinical trials examining the use of tamoxifen for a period greater than 5-years demonstrate that a subset of ER + breast cancer patients benefit from such treatment [20]. Hence, patients with subgroup #3 tumors might represent those who benefit from extended tamoxifen treatment.

As additional validation, we investigated the prevalence of the 6 subgroups in the TCGA breast data set, which comprised 801 ER + breast tumors [4]. Using the PAM classifier described above, the mean probability for classification was 86%, and more than 70% (n = 580) of the tumors in the TCGA set were assigned a probability of 80% or higher of belonging to one of the 6 subgroups (Additional file 2: Figure S5 A&B). Hence, this extra validation data set provides additional evidence for the robustness of the classification framework.

Taken together with our previous data, these results demonstrate that the 6 identified subgroups of ER + breast tumors can be reproducibly identified in independent patient cohorts and provide a clinically relevant means of classifying ER + breast tumors.

### ER + subgroups enable predictive modeling of anti-cancer drug sensitivity

**Figure 4:**
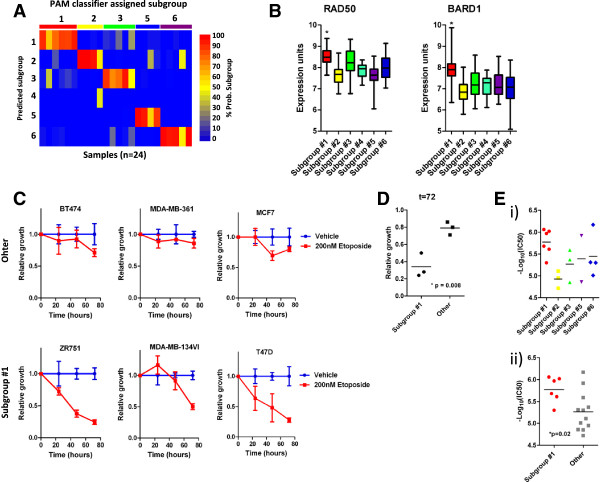
**Subgroup specific response to anti-cancer compounds. A)** Cell line subgroup assignment based on PAM classifier. **B)** Expression of RAD50 and BARD1 in the 6 subgroups subgroups. **C)** Relative growth analysis of subgroup 1 and non-subgroup #1 cell with or without 200nM etoposide. **D)** Relative growth at 72 hours for subgroup #1 and other cell lines reveals marked etoposide selectivity for subgroup #1 cell lines. **E)** Cell line sensitivity to etoposide from the Heiser *et al*. published dataset (*p = 0.02, t-test).

As described above, the established framework allows classification of ER + breast tumors into 1 of 6 subgroups based on patterns in estrogen independent gene expression. We first tested whether this framework could be extended to classify ER + breast tumor cell lines into the same subgroups. We accessed previously described breast tumor cell line gene expression datasets [6, 13] and classified ER + breast tumor cell lines into the 6 subgroups. Among 24 ER + breast tumor cell lines, 5 of the 6 subgroups were represented by at least 4 cell lines, thus providing experimental models for these subgroups (Figure 4A, Additional file 1: Table S2). We sought to identify compounds with subgroup specificity for the most aggressive subgroups based on our analyses of DMFS in the patient cohorts. Although patients with subgroup #4 tumors experienced the worst outcome, we failed to identify any subgroup #4 cell lines. Hence we focused our efforts on the second most aggressive subgroup, which was subgroup #1. We observed that subgroup #1 tumors tended to over-express genes involved in the repair of double stranded (ds) DNA breaks, including RAD50 [21] and BARD1 [22], suggesting that subgroup #1 tumors possess a ‘dsDNA break’ phenotype and may be hypersensitive to agents that induce dsDNA breaks (Figure 4B). We also examined RAD50 and BARD1 expression in cell lines stratified by subgroup, however this analysis was inconclusive likely owing to the fact that most subgroups comprised very few lines (Additional file 2: Figure S6A). Subgroup #1 cell lines were hypersensitive to etoposide, a potent and specific inducer of dsDNA breaks [23] (Figure 4C). Specifically, we compared the relative growth (to control) of 3 subgroup #1 cell lines and 3 cell lines belonging to other subgroups over 72 hours of treatment with 200nM etoposide. After 72 hours, relative growth was significantly lower in subgroup #1 cell lines (34% of control) compared to the relative growth of non-subgroup #1 cell lines (79% of control)(Figure 4D, *p = 0.008, t-test). To confirm these findings, we obtained breast tumor cell line drug sensitivity data that was previously reported by Heiser *et al*. in 2012 [6], for 18 cell lines that were also present within the cell line gene expression data set. Similar to our previous observations, we observed the subgroup #1 cell lines were generally the most sensitive to etoposide (Figure 4E-i. The mean –log_10_(IC50) of subgroup #1 cell lines was significantly lower than cell lines belonging to other subgroups (Figure 4E-ii, *p = 0.02, t-test).

To extend these findings, we looked for over-expression of other actionable targets with subgroup selective expression among the 6 subgroups (Additional file 2: Figure S6). IGF2 was over-expressed in subgroup 2 tumors, implicating IGF signaling as a therapeutic target in subgroup#2 tumors. Interestingly, subgroup #3 tumors significantly over-expressed the angiotensin receptor 2 (AGTR2). Whereas AGTR2 hasn’t been a target for cancer drug development, it has been a successfully exploited target for the development of hypertension drugs [24]. Other notable targets included over-expression of the anti-apoptotic protein BCL2 in subgroup #3 tumors, and the immune-modulatory target CTLA4 in subgroup #5 tumors. In each case, approved therapeutics exist or are under development that target these highlighted over-expressed genes. These observed patterns could potentially be used to target therapies in ER + breast cancer patients contingent on the subgroup membership of their tumor.

## Discussion

Substantial molecular heterogeneity exists among ER + tumors, which isn’t adequately captured by either histophathological variables or more recent molecular subtyping strategies. Accordingly, we sought to identify novel means of classifying ER + tumors, and reproducibly identified 6 subgroups of ER + tumors based on the expression of estrogen independent genes. Notably, we also observed survival and treatment sensitivity differences among the 6 subgroups. Hence, our data suggests that patient subgroup membership may be a useful tool for guiding treatment of ER + breast cancer patients.

Briefly, the subgroup identification strategy was highly similar to that originally described by Perou *et al*. in 2000 [1]. Whereas Perou *et al*. employed an unsupervised clustering approach with intrinsic genes in unselected breast tumors, we employed unsupervised clustering with estrogen independent genes in breast tumors selected for ER positivity. For this experiment we analysed gene expression profiling data from 58 ER + tumors biopsied from post-menupausal women before and after treatment with letrozole [7]. We hypothesized that genes whose expression showed minimal variation after letrozole treatment could be considered to be expressed independent of estrogen, and identified estrogen independent genes based on this assumption. However, many breast cancer patients are pre-menopausal and receive different endocrine therapies for breast cancer treatment, namely tamoxifen. It is unclear whether the definition of estrogen independent genes we propose here would be different in pre-menopausal patients, or patients treated with alternate endocrine agents, and these possibilities represent intriguing avenues for future research. We note however, that subgrouping ER + tumors based on estrogen independent gene expression was both robust and reproducible in cohorts of tumors that included pre-menopausal patients as well as those treated with tamoxifen, suggesting that our approach is broadly applicable.

There remain several limitations of the work reported herein. All of our conclusions are based on the analysis of retrospective data, which limits its clinical value. We validated the occurrence, and clinical attributes, of the 6 subgroups in relatively large independent cohorts, however a true estimate of the clinical usefulness of the 6 subgroup classification for ER + breast cancers would require additional validation in clinical trial samples, as well as completion of a prospective clinical trial examining the capacity of the classification to guide therapy. In addition, it isn’t clear if subgroup classification would add meaningful clinical information beyond that obtained from existing prognostic tests designed for ER + tumors, such as OncotypeDX® [25]. For example, a relevant question that remains is whether the good prognosis subgroups identified here (subgroups 2,5&6) experience similarly excellent survival to the low risk group identified by OncotypeDX®. Additionally, it isn’t clear if the relationship between patient outcome and subgroup assignment is a consequence of subgroup association with natural progression of breast cancer or tumor response to adjuvant endocrine therapy. Many of the patients obtained from publically available sources had incomplete clinical annotations, and they comprise a mixture of patients that received no adjuvant therapy, or adjuvant tamoxifen, likely lasting for 5 years. Based on these data it is difficult to discern how differences in extent or choice of endocrine therapy might influence the relationship between patient outcome and subgroup membership. Hence, although our data suggests the 6 subgroup classification of ER + breast cancer may be useful for guiding therapy in patients, many additional validation experiments are required to confirm our findings.

## Conclusion

Ultimately, we propose that the 6 subgroups described here provide a strategy for improved understanding and treatment of ER + breast tumors. We demonstrate that the subgroups are unique and independent of the molecular subtypes of cancer, and provide a clinically relevant means of tumor classification. We anticipate that subgrouping will provide a framework to both guide optimal use of existing therapeutics, as well as gain insight into biological processes that represent relevant targets for development of the next generation of experimental therapies.

## Electronic supplementary material

Additional file 1: **Supplemental tables.** (XLSX 52 KB)

Additional file 2: **Supplemental figures.** (PPTX 900 KB)
