# Supplementary material for: Estrogen independent gene expression defines clinically relevant subgroups of estrogen receptor positive breast cancer
Source: BMC Cancer. 2014 Nov 24;14:871. doi: 10.1186/1471-2407-14-871 (PMC4289221; doi:10.1186/1471-2407-14-871)
Supplement: Supplementary file 2 — Additional file 2: Supplemental figures. (PPTX 900 KB) [file 12885_2014_5122_MOESM2_ESM.pptx]

## Slide 1
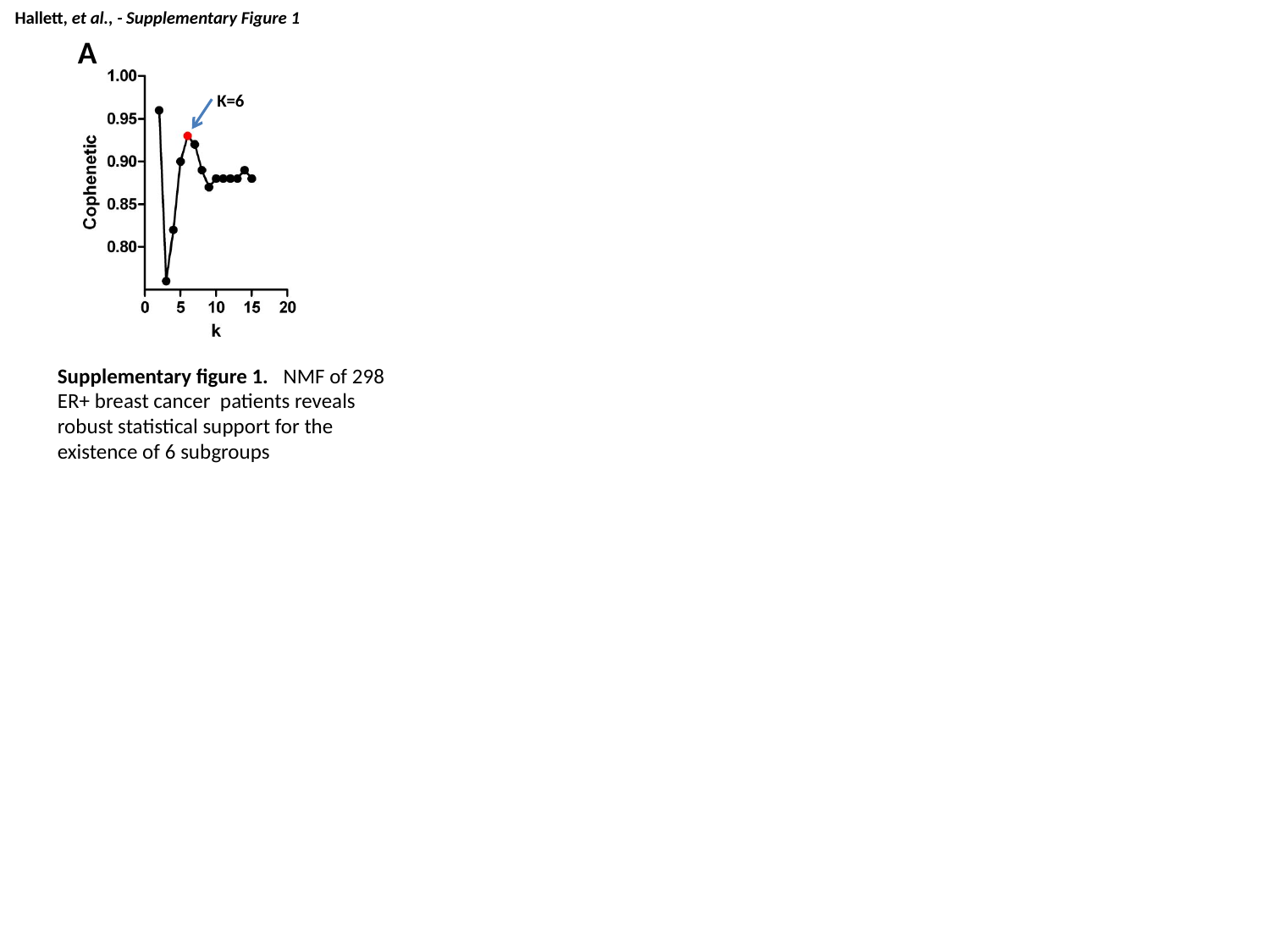

Hallett, et al., - Supplementary Figure 1
A
K=6
Supplementary figure 1. NMF of 298 ER+ breast cancer patients reveals robust statistical support for the existence of 6 subgroups

## Slide 2
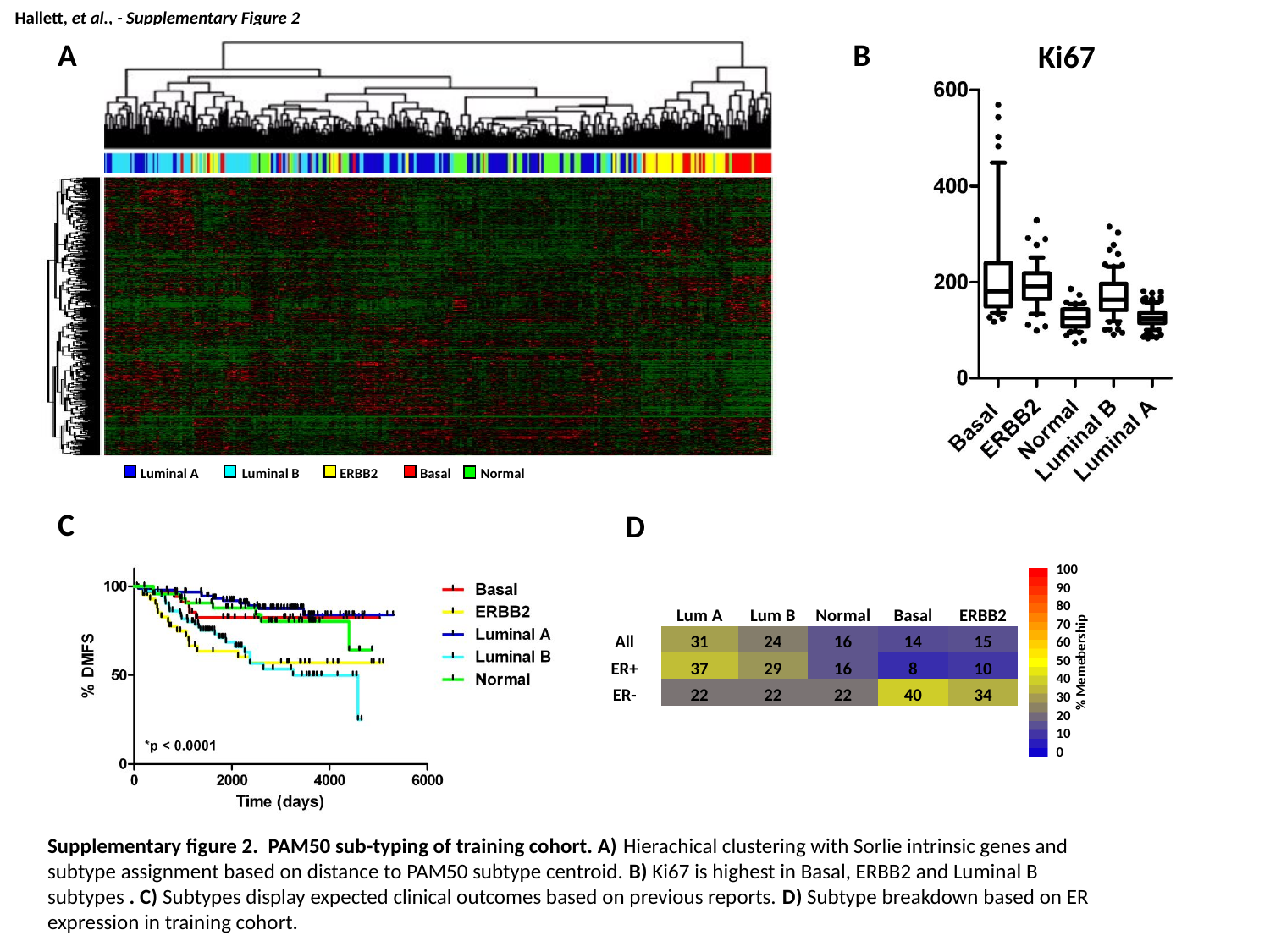

Hallett, et al., - Supplementary Figure 2
A
B
Ki67
Luminal A
ERBB2
Basal
Normal
Luminal B
C
D
| 100 |
| --- |
| 90 |
| 80 |
| 70 |
| 60 |
| 50 |
| 40 |
| 30 |
| 20 |
| 10 |
| 0 |
| | Lum A | Lum B | Normal | Basal | ERBB2 |
| --- | --- | --- | --- | --- | --- |
| All | 31 | 24 | 16 | 14 | 15 |
| ER+ | 37 | 29 | 16 | 8 | 10 |
| ER- | 22 | 22 | 22 | 40 | 34 |
% Memebership
Supplementary figure 2. PAM50 sub-typing of training cohort. A) Hierachical clustering with Sorlie intrinsic genes and subtype assignment based on distance to PAM50 subtype centroid. B) Ki67 is highest in Basal, ERBB2 and Luminal B subtypes . C) Subtypes display expected clinical outcomes based on previous reports. D) Subtype breakdown based on ER expression in training cohort.

## Slide 3
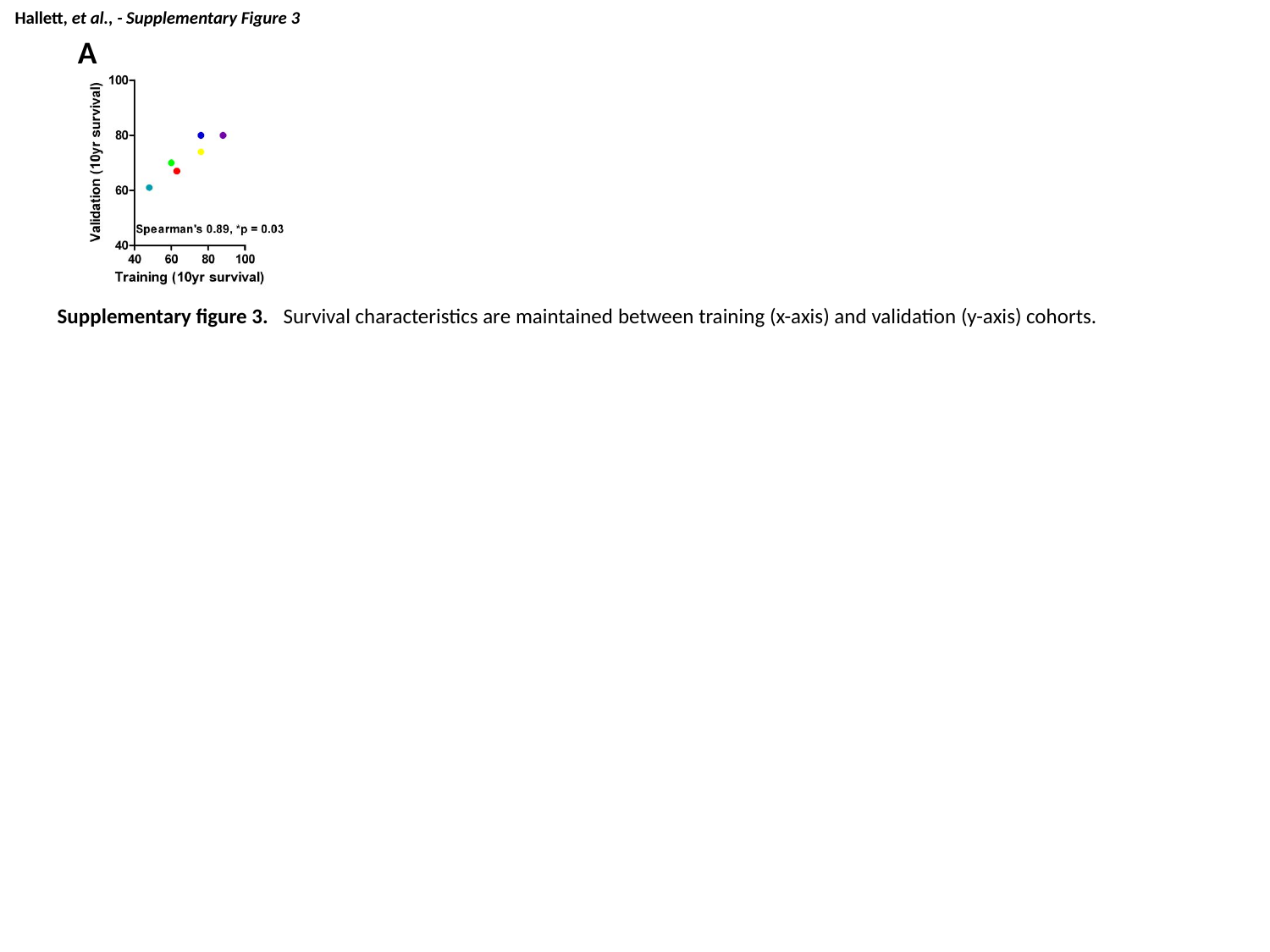

Hallett, et al., - Supplementary Figure 3
A
Supplementary figure 3. Survival characteristics are maintained between training (x-axis) and validation (y-axis) cohorts.

## Slide 4
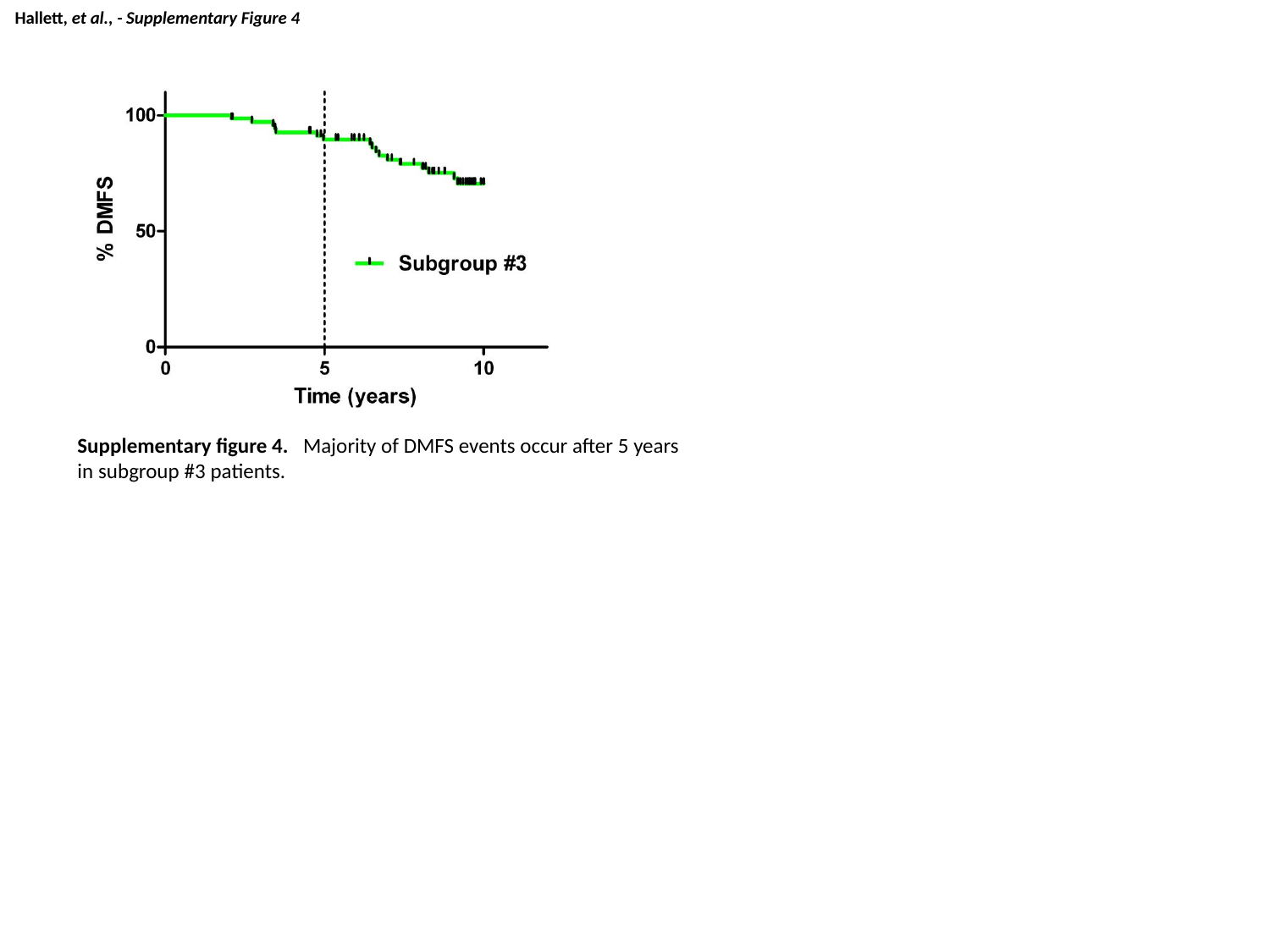

Hallett, et al., - Supplementary Figure 4
Supplementary figure 4. Majority of DMFS events occur after 5 years in subgroup #3 patients.

## Slide 5
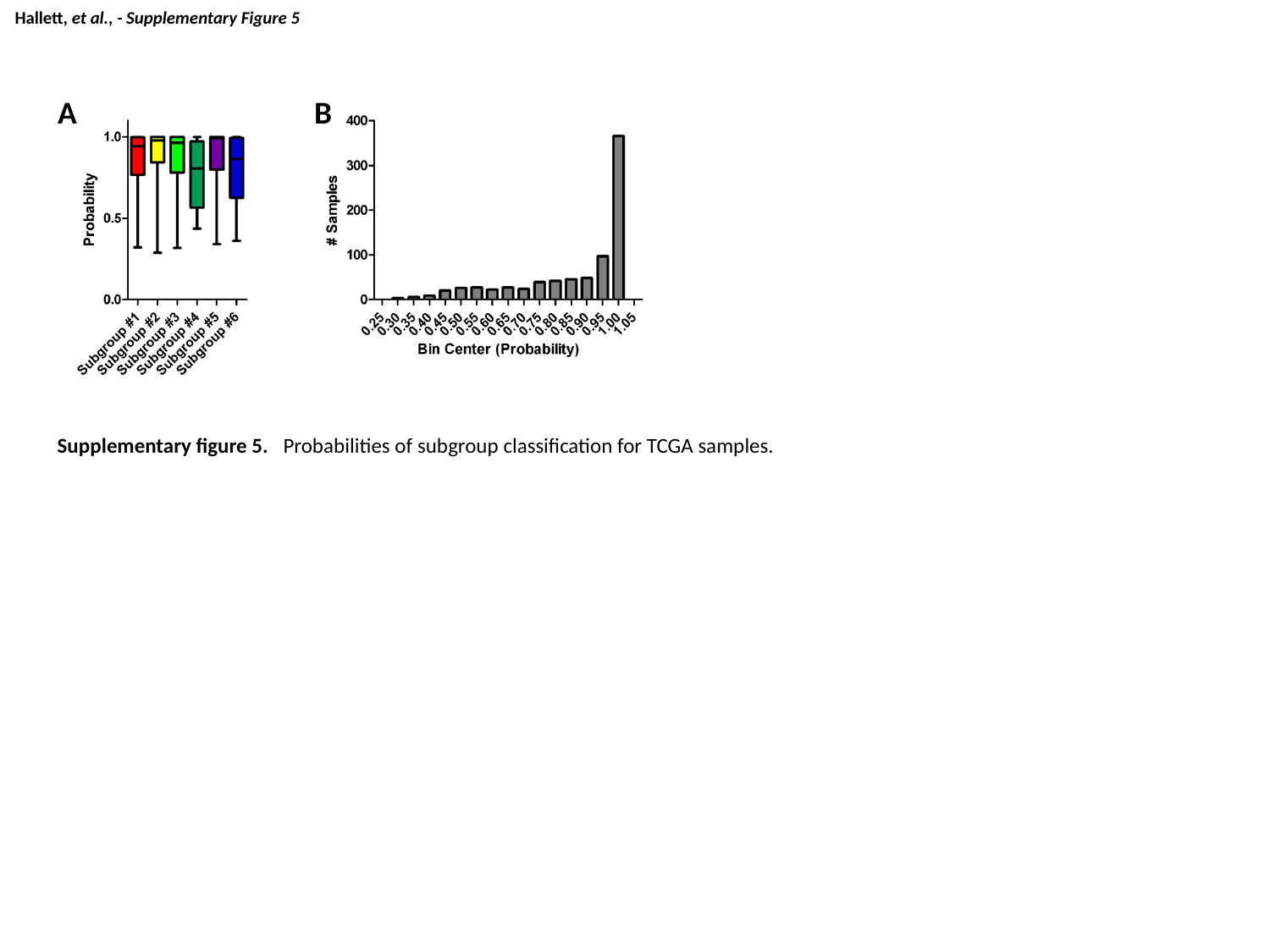

Hallett, et al., - Supplementary Figure 5
A
B
Supplementary figure 5. Probabilities of subgroup classification for TCGA samples.

## Slide 6
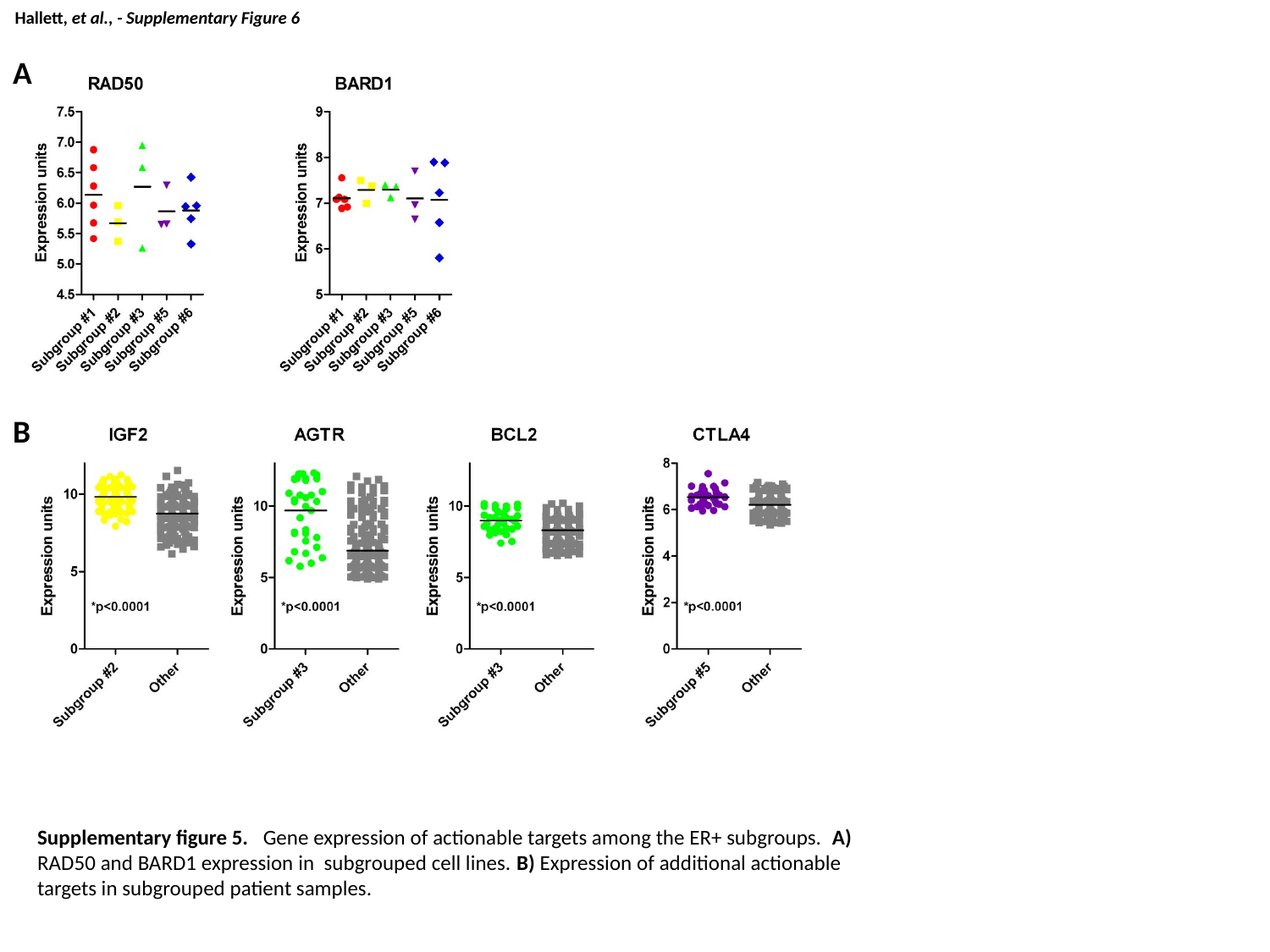

Hallett, et al., - Supplementary Figure 6
A
B
Supplementary figure 5. Gene expression of actionable targets among the ER+ subgroups. A) RAD50 and BARD1 expression in subgrouped cell lines. B) Expression of additional actionable targets in subgrouped patient samples.
